# Shaoxia: a web-based interactive analysis platform for single cell RNA sequencing data

**DOI:** 10.1186/s12864-024-10322-1

**Published:** 2024-04-24

**Authors:** Weideng Wei, Xiaoqiang Xia, Taiwen Li, Qianming Chen, Xiaodong Feng

**Affiliations:** 1 State Key Laboratory of Oral Diseases & National Center for Stomatology & National Clinical Research Center for Oral Diseases & Research Unit of Oral Carcinogenesis and Management & Chinese Academy of Medical Sciences, West China Hospital of Stomatology, Sichuan University, No. 14, 3rd Section of Ren Min Nan Rd., Chengdu, Sichuan 610041 China; 2 Key Laboratory of Oral Biomedical Research of Zhejiang Province, Affiliated Stomatology Hospital, Zhejiang University School of Stomatology, Hangzhou, Zhejiang 310006 China

**Keywords:** Single cell RNA sequencing, Analysis framework, Pipeline, Analysis platform

## Abstract

**Background:**

In recent years, Single-cell RNA sequencing (scRNA-seq) is increasingly accessible to researchers of many fields. However, interpreting its data demands proficiency in multiple programming languages and bioinformatic skills, which limited researchers, without such expertise, exploring information from scRNA-seq data. Therefore, there is a tremendous need to develop easy-to-use software, covering all the aspects of scRNA-seq data analysis.

**Results:**

We proposed a clear analysis framework for scRNA-seq data, which emphasized the fundamental and crucial roles of cell identity annotation, abstracting the analysis process into three stages: upstream analysis, cell annotation and downstream analysis. The framework can equip researchers with a comprehensive understanding of the analysis procedure and facilitate effective data interpretation. Leveraging the developed framework, we engineered Shaoxia, an analysis platform designed to democratize scRNA-seq analysis by accelerating processing through high-performance computing capabilities and offering a user-friendly interface accessible even to wet-lab researchers without programming expertise.

**Conclusion:**

Shaoxia stands as a powerful and user-friendly open-source software for automated scRNA-seq analysis, offering comprehensive functionality for streamlined functional genomics studies. Shaoxia is freely accessible at http://www.shaoxia.cloud, and its source code is publicly available at https://github.com/WiedenWei/shaoxia.

**Supplementary Information:**

The online version contains supplementary material available at 10.1186/s12864-024-10322-1.

## Background

In recent years, single cell sequencing technologies emerge as a revolutionary way to interrogate the essential biological issues, from tissue development and homeostasis to disease specific mechanisms. Harnessing single cell analyses, researchers can reveal new and potentially unexpected biological discoveries relative to traditional profiling methods that assess bulk populations [1]. Single cell technologies have gained a tremendous advance within the last decade. Among these approaches, single cell RNA sequencing (scRNA-seq) stands out as the most popular and mature method. Advancements in technology and cost reduction have made scRNA-seq increasingly accessible to researchers in a broad spectrum of fields. For instance, scientists have employed scRNA-seq to identify new cell subtypes [2], investigate immunological responses to COVID-19 (corona virus disease 2019) [3], profile the immune cell landscape of metastatic tumors [4], comprehend early embryonic development [5], contribute to the construction of the International Human Cell Atlas [6], pinpoint new cancer cell subtypes [7], delineate kinetics during the progression of B cell acute lymphoblastic leukemia [8], and delve into the formation of the immunosuppressive tumor microenvironment [9], among other applications.

Despite the fact that scRNA-seq is widely used in a variety of research fields, it also brings new challenges in data analysis aspect. Firstly, there is not a generally accepted analysis framework for scRNA-seq data, although several computational pipelines for single cell sequencing data exist. For example, Hwang et.al. proposed an analysis framework, which emphasized on solving the problem of noisy scRNA-seq data [1]. Baek et.al. came up with another one for scATAC-seq (single-cell sequencing assay for transposase-accessible chromatin) data, which consists of pre-processing and downstream analysis [10]. However, a well-structured analysis framework with a high level of abstractness can assist researchers in conducting more effective data interpretation. Secondly, in comparison to bulk mRNA sequencing methods, scRNA-seq introduces a new dimension (single-cell resolution) to RNA sequencing data, resulting in significantly larger raw sequencing data—typically tens of gigabases per sample. Handling such “big data” demands substantial computational resources, making it impractical for personal computers or small server setups to execute these data processing tasks. Thirdly, many currently published single-cell analysis tools, such as Seurat [11], CellphoneDB [12], and SCENIC [13], demand an advanced level of programming proficiency, involving mastery of multiple programming languages. Consequently, these tools are not user-friendly and accessible to researchers with limited or no programming skills, despite their professional expertise in biology. In other words, there is a tremendous need to develop a platform, covering all the aspects of scRNA-seq data analysis.

Herein, we propose an intuitive and clear framework for scRNA-seq data. In this framework, we emphasize the fundamental and crucial roles of cell identity annotation, abstracting the analysis process into three stages: upstream analysis, cell annotation and downstream analysis. Moreover, having this framework in hand, we designed and developed a web-based, interactive analysis platform, named Shaoxia, that releases the power of modern compute system (high performance compute, HPC) to accelerate the analysis of scRNA-seq data and makes aspects of single cell RNA-seq data analysis friendlier and more accessible for researchers, especially who focus on wat-lab technologies and has no programming skills. Shaoxia employs a set of popular tools as backend to enable robust data analysis. To demonstrate Shaoxia's functionalities and compatibility, we employed it to analyze scRNA-seq datasets from both a standard PBMC (peripheral blood mononuclear cell) sample and real-world oral mucosa tissue (healthy gingival mucosa, GM, and gingival mucosa from periodontitis, PD).

## Implementation

### Analysis framework for scRNA-seq data

**Fig. 1:**
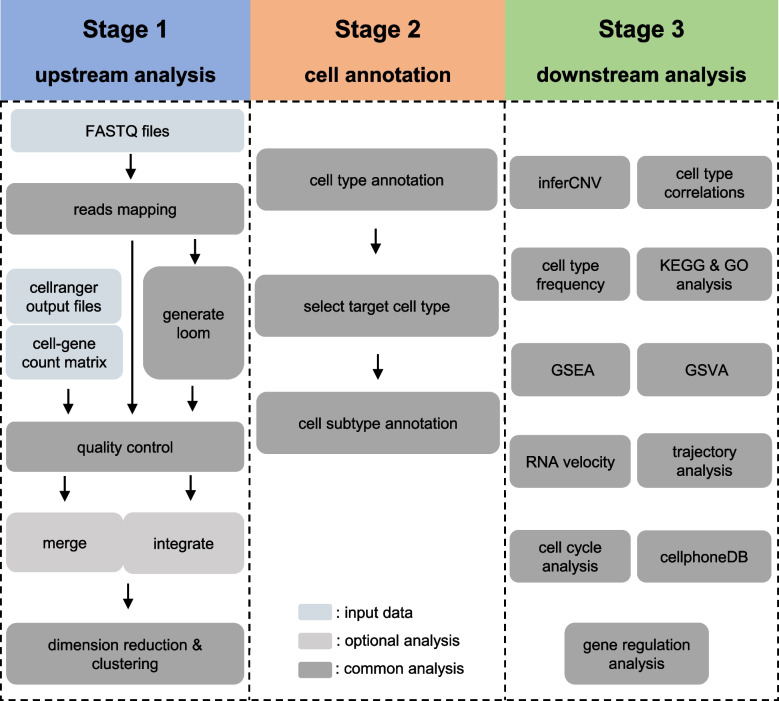
Analysis framework for scRNA-seq data

Cell annotation is fundamental part of scRNA-seq data analysis, without cell identity, analyses such as differentially expressed genes (DEGs) analysis have no biological means, or even worse, it may lead to wrong conclusions when based on incorrect cell annotation. With this understanding in mind, we propose an intuitive and clear framework that upstream and downstream analysis are separated by cell type annotation (Fig. 1), emphasizing the important roles of cell annotation.

The major step of upstream analysis contains reads mapping, cell quality control, data combining, dimensional reduction and cell clustering. Starting with FASTQ files, reads mapping should be the first analysis, it maps the reads to the reference genome, and then generates the expression matrix. The rows of the matrix are the gene names, and the columns are the cell names. After reads mapping, a special process step – generate loom can be conducted, it can generate essential files for RNA velocity analysis. With expression matrix, cell quality control can be conducted, this process filters out expression data from dead cells and cells that have low quality. After quality control, expression data from multiple samples can be merged if they are same sample type and have same experiment design, if they are not the same sample type or same experiment design, their expression data can also be integrated. In our analysis framework, data combining is optional. Because of sparse nature of expression data generated by current single cell RNA sequencing technology, the last two step of upstream analysis – dimension reduction and cell clustering should be carried out, it can reduce the high dimension of expression matrix (thousands of genes, thus thousands of dimensions), the cells are then clustered, subsequent cell annotation will be carried out based on these cell clusters.

As mentioned above, cell annotation is the most important and fundamental part of scRNA-seq data analysis. In our analysis framework, cell annotation has two major parts – cell type annotation and cell subtype annotation, this design follows the recommendations of a cell annotation protocol with some modifications [14]. First, annotation begins by identifying major well-known cell types which have generally acknowledged cell markers, for example, with scRNA-seq data from blood samples, T cells are easy to identify by *CD3D* and *CD3E* gene markers, B cells can also be easy to identify by *MS4A1* and *CD79A* gene markers. Second, to identify subtypes within the major cell types, cells belonging to the targeted major cell population are re-clustered and subsequently annotated using reference gene markers from the literature or CellMarker database [15], this process enable us to identify cell subtypes and even new cell subtypes. For instance, *CD8*^+^ or *CD4*^+^ T cells can be identified from its major cell type T cell, memory B cells or naïve B cells can be identified from its major cell type B cell. The annotation procedure of our framework may be better than annotating cell subtypes directly, because the procedure – annotating major cell types and then cell subtypes could be easier and avoid potential annotation errors.

In our framework, eleven downstream analyses fall into two major fall into two major classes.: 1) gene level analysis, including DEGs analysis, KEGG (kyoto encyclopedia of genes and genomes) and GO (gene ontology) enrichment analysis of DEGs, gene set enrichment analysis (GSEA), gene set variation analysis (GSVA), inferred copy number variation (inferCNV), gene regulation analysis; 2) cell level analysis, which contains cell type correlation, cell cycle scoring, cell type frequency, RNA velocity, pseudo-time inference, cell communication analysis. Our framework covers a wide range of downstream analysis, which can meet the needs of most researchers.

### Implementation of Shaoxia platform

**Fig. 2:**
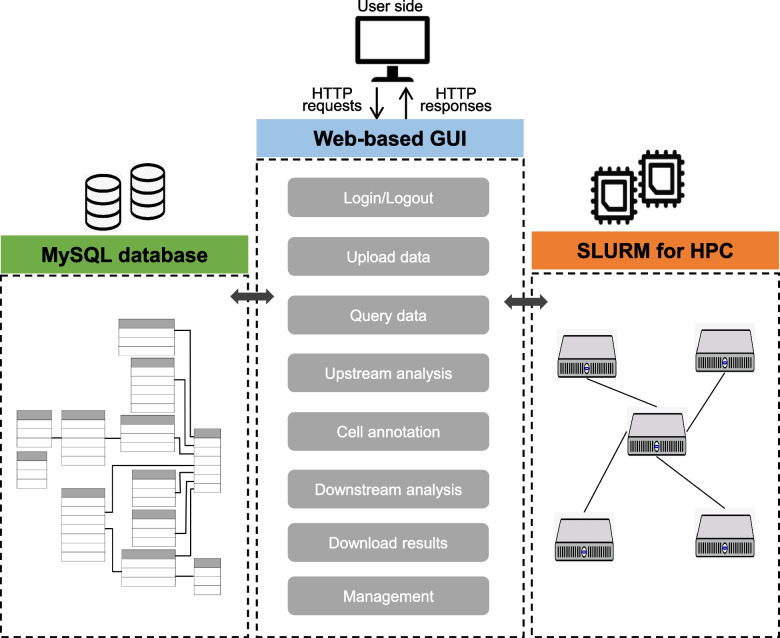
Architecture of Shaoxia platform

Based on the scRNA-seq data analysis framework, we designed and developed a web based graphical user interface (GUI) platform (Fig. 2), named Shaoxia, which enable researchers to easily explore and interpret their scRNA-seq data. Shaoxia supports three type of data format as input: 1) 10X Genomics cellranger software output data files (barcodes.tsv.gz, features.tsv.gz and matrix.tsv.gz), 2) FASTQ files that generated by 10X Genomics single cell RNA sequencing protocol, 3) cell-gene count matrix that is a tab-delimited text file, the first row contains cell barcodes, and the first column contains gene names, each entry in the matrix represents the molecule count for a specific cell-gene pair. These three types of data files can be directly uploaded to Shaoxia platform. Besides, Shaoxia integrates all kinds of analysis tool and covers diverse aspects of scRNA-seq data analysis (Table 1).

**Table 1:** Summary of analysis tools used in Shaoxia

Stage	Step	Command	Software tool
**Upstream** **analysis**	Reads mapping	cellranger count	10X Genomics cellranger
	Generate loom	samtools sort velocyto run10x	Samtools [16] scvelo Python package [17]
	Quality control	Seurat::PercentageFeatureSet	Seurat R package [11]
	Integration	Seurat::FindIntegrationAnchors	Seurat R package [11]
	Merge	Seurat::merge	Seurat R package [11]
	Dimension reduction & clustering	Seurat::FindVariableFeatures Seurat::NormalizeData Seurat::ScaleData Seurat::RunPCA Seurat::FindNeighbours Seurat::FindClusters Seurat::RunUMAP	Seurat R package [11]
**Cell** **annotation**	Cell type annotation	-	Seurat R package [11]
	Cell subtype annotation	-	Seurat R package [11]
**Downstream** **analysis**	inferCNV	infercnv::run	infercnv R package [18]
	Cluster correlation	ComplexHeatmap:: Heatmap	ComplexHeatmap R package [19]
	Cell cycle scoring	Seurat::CellCycleScoring	Seurat R package [11]
	Cell type frequency	ggplot2::geom_bar	ggplot2 R package [20]
	KEGG & GO erichment	Seurat::FindMarkers ClusterProfiler::enrichKEGG ClusterProfiler::enrichGO	Seurat R package [11] ClusterProfiler [21]
	GSVA	gsva:: gsva	gsva R package [22]
	GSEA	gsea-cli.sh GSEA	GESA software [23]
	Trajectory anlysis	monocle::setOrderingFilter monocle:: reduceDimension monocle:: orderCells monocle3::cluster_cells monocle3::learn_graph monocle3::order_cells	Monocle and Monocle3 R package [24]
	RNA velocity	velocity_embedding_stream	scvelo Python package [17]
	Cell communiction	cellphonedb method cellphonedb plot	cellphonedb Python package [12]
	Gene regulation	pyscenic grn pyscenic ctx pyscenic aucell	SCENIC Python package [13]

In upstream analysis stage, reads mapping is performed by 10X Genomics cellrager software. In generate-loom step, the loom file is generated by samtools [16] and scvelo Python package [17] and is used in RNA velocity downstream analysis. Quality control (QC) has four metrics that are calculated by Seurat [11] function PercentageFeatureSet, the visualization images of QC effect are produced by Seurat [11] function VlnPlot. Extremely low number of detected genes(nFeature_RNA) per-cell could indicate loss-of-RNA, in contrast, extremely high number of detected genes per-cell could indicate doublets, and low number of molecule counts (nCount_RNA) per-cell could indicate low quality of data, high percentage of mitochondrial gene (percent.mt) expression could indicate dying cells, extremely low proportion of ribosomal gene (percent.rp) expression could indicate loss-of-RNA. If users want to combine multiple sample’s data, Shaoxia provides two type of methods – merge and integrate, both of them come from Seurat R package [11]. In dimension reduction and clustering step, some Seurat package [11] functions are used, including NormalizeData, FindVariableFeatures, ScaleData, RunPCA, RunUMAP, FindNeighbors, FindClusters and Dimplot.

In cell type annotation stage, Markers of all cell clusters are generated by Seurat [11] function FindAllMarkers, marker plots and cell type annotation result images are produced by Seurat [11] function FeaturePlot, DoHeatmap, VlnPlot.

In downstream analysis stage, inferCNV is carried out by infercnv R package [18], cell type correlations are analyzed by R function cor and its result image is produced by using R package ComplexHeatmap [19]. Cell type frequency is computed by custom R function and visualized by functions of ggplot2 [20]. DEGs is generated by Seurat [11] function FindMarkers, KEGG and GO enrichment analysis is performed by R package ClusterProfile [21], GSVA analysis is carried out by GSVA R package [22], GSEA is performed using GSEA software [23]. RNA velocity is carried out by scvelo Python package [17], cell trajectory analysis is performed with Monocle and Monocle3 R package [24], cell communication analysis results are produced by CellPhoneDB [12] Python package, gene regulative analysis results are generated by SCENIC [13] Python package.

In term of data management, Shaoxia has a data-free design, a special MySQL database is established to save the parameters users submitted rather than the intermediate files produced during the analysis, thus only analysis (images) results and upload data are stored typically. Furthermore, Shaoxia employs SLURM software to manage analysis jobs on HPC system. At last, all the results of upstream, cell type annotation and downstream analysis can be directly downloaded from the platform.

### Processing scRNA-seq data from PBMC and oral mucosa using Shaoxia

Raw sequencing files (FASTQ) of PBMC scRNA-seq data (two sample, s1 and s2) were downloaded from archive data website (http://s3-us-west-2.amazonaws.com/10x.files/samples/cell-exp/2.1.0/pbmc8k/pbmc8k_fastqs.tar) and uploaded to the platform. The quality control parameters are set to 200 < nFeature_RNA < 4000, percent.mt < 15, nCount_RNA > 2000 and percent.rp > 20. Two samples’ data are integrated together. Dimension reduction and cell cluster use first 10 principal components and 0.5 resolution. In downstream analysis, related parameters are set to default.

Cellranger output files of oral tissues (GM and PD) were downloaded via the following link: https://www.ncbi.nlm.nih.gov/geo/query/acc.cgi?acc=GSE164241. Six samples’ data (including GM148, GM169, GM283, PD170, PD164b and PD164) was uploaded to the platform. The quality control parameters are set to 200 < nFeature_RNA < 6000, percent.mt < 15, nCount_RNA > 2000 and percent.rp > 0. Six samples’ data were integrated together. Dimension reduction and cell cluster use first 10 principal components and 0.1 resolution. In term of B cell sub-clustering, dimension reduction and cell cluster use first 8 principal components and 0.1 resolution. In downstream analysis, related parameters are set to default.

## Results and discussion

### Perform PBMC scRNA-seq data analysis with Shaoxia

To demonstrate the core functionality of the Shaoxia scRNA-seq data analysis platform, we analyzed a publicly available dataset that profiled messenger RNA abundance of PBMCs, generated by 10 × Genomics. These PBMC datasets is often used to benchmark all kinds of bioinformatic software. The data we use contains two samples – s1 and s2, each one has approximately eight-thousands of cells, and this dataset was utilized for upstream analysis, cell annotation, and downstream analysis.

**Fig. 3:**
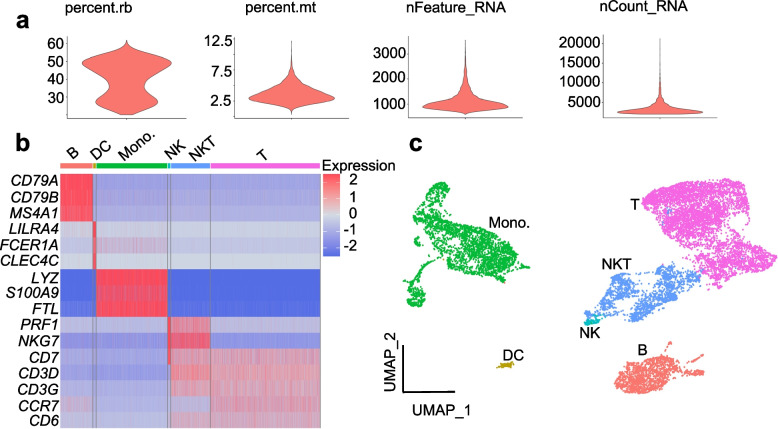
Results of quality control and cell annotation of PBMC scRNA-seq data. **a** quality control result. **b** heatmap plot of cell type specific gene marker expression level. **c** cell clustering and annotation result

After reads mapping and generate-loom, four quality control metrics are used, including the number of expressed genes (nFeature_RNA), the number of unique molecular identifier (UMI) counts (nCount_RNA), the percentage of all the counts belonging to mitochondrial genes (percent.mt), the percentage of all the counts belonging to ribosomal genes (percent.rb) (Fig. 3a) to remove the low-quality cells and exclude doublet, resulting in a dataset of total 15,446 cells. After checking potential gene markers, we identified six cell types – B cell, dendritic cell (DC), monocyte, NK (natural killer) cell, NKT cell and T cell (Fig. 3b and c).

**Fig. 4:**
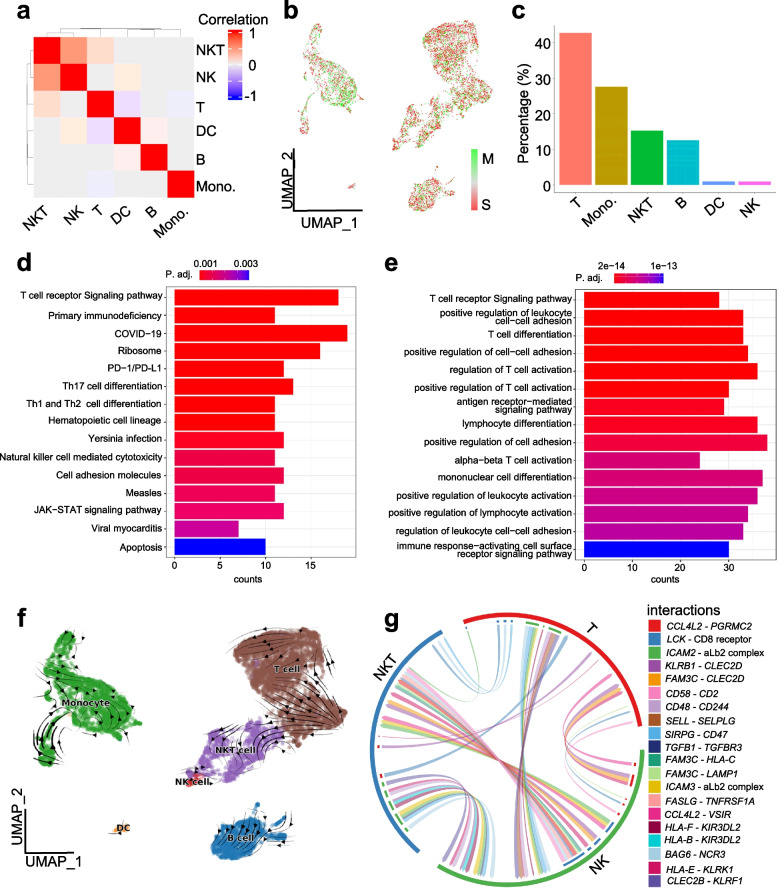
Downstream analysis results of PBMC scRNA-seq data. **a** cell type correlation. **b** cell cycle analysis. **c** cell type frequency. **d** KEGG enrichment result of DEGs of T cell versus B cell. **e** GO enrichment result of DEGs of T cell versus B cell. **f** RNA velocity analysis result. **g** cell communication analysis result

In cell type correlation analysis result, NKT cell and NK cell have close relationship, correlation among other cell types is weak, this demonstrate that our cell type annotation has high credibility (Fig. 4a). Cell cycle scoring analysis show that most of cells stay at S phase, minor of cells stays at M phase (Fig. 4b). According to cell type frequency analysis, T cell has highest frequency (42%) while DC and NK cell have lowest frequency (Fig. 4c). To compare T cell with B cell, DEGs analysis, KEGG and GO enrichment analysis are performed. The KEGG enrichment result show that DEGs of T cell and B cell relate to immune pathways, such as T cell receptor signaling pathway and primary immunodeficiency (Fig. 4d), this is consistence with the GO enrichment results (Fig. 4e), GSEA results (Figure S1) and GSVA result (Figure S2). RNA velocity analysis show that B cell has a single direction of state transition, while monocyte, T cell and NKT cell have multiple direction of state transition. Especially, NKT cell and T cell have interaction of state transition (Fig. 4f). In cell communication analysis, we note that there are many interactions between NK cell, NKT cell and T cell. Especially for *LCK-CD8* interaction between NKT cell and T cell, it indicates that the function of NKT cell may be regulated by *CD8*^+^ T cells (Fig. 4g). In gene regulation analysis, we found that monocytes have a very high transcription factors activity level, when compared to other cell types, it may indicate that there is rapid state transition among monocytes. Transcription factor *LEF1* seem to have activity in all the cell types, it suggests that *LEF1* may play a role in regulating different cell types (Figure S3).

### Analyzing oral tissue scRNA-seq dataset using Shaoxia

Beyond standard PBMC data processing, a real-world scRNA-seq dataset from healthy gingival mucosa (GM) and mucosa from individuals with periodontitis (PD) was analyzed on Shaoxia platform, aim to elucidate the molecular underpinnings of gingival health and disease.

**Fig. 5:**
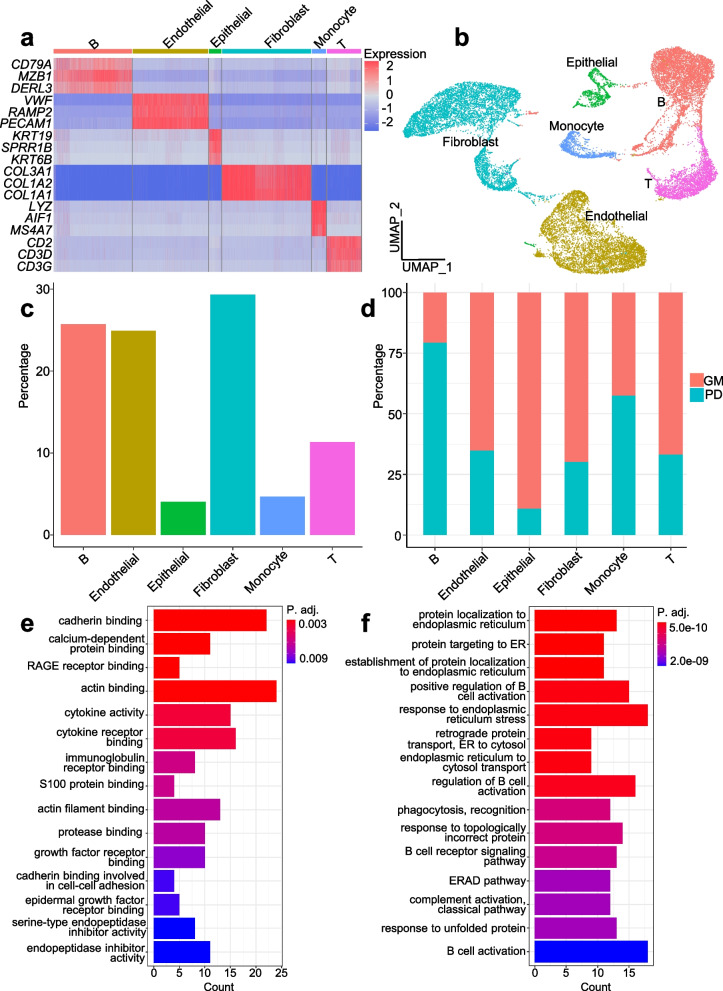
Analysis results of oral tissue scRNA-seq data. **a** heatmap plot of cell type specific gene marker expression level. **b** cell clustering and annotation result. **c** overall cell type frequency. **d** sample type specific cell type frequency. **e** GO enrichment result of DEGs of PD versus GM in epithelial cell. **f** GO enrichment result of DEGs of PD versus GM in B cell

**Fig. 6:**
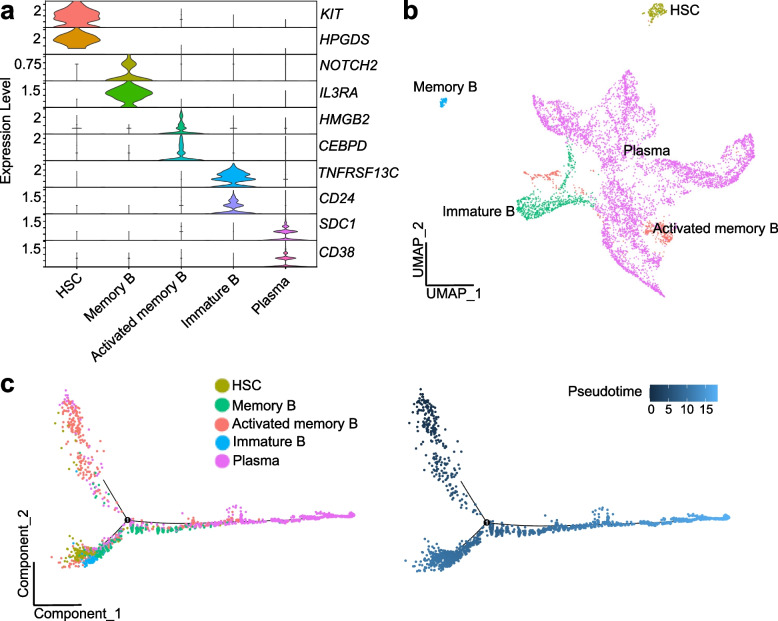
B cell subtypes analysis results of oral tissues. **a** stack violin plot of B cell subtype specific gene marker expression level. **b** B cell subtypes clustering and annotation result. **c** trajectory and pseudotime analysis results

After quality control, 24,541 cells remain, and six cell types are identified—B cell, endothelial cell, epithelial cell, fibroblast, monocyte and T cell (Fig. 5a and b). In cell frequency analysis result, it shows that epithelial cell has lowest frequency, while B cell has relatively high frequency (Fig. 5c). Interestingly, GM has much higher frequency in epithelial cell, while PD has much higher frequency in B cell (Fig. 5d). Therefore, we performed enrichment analysis specifically focusing on epithelial cell and B cell, to compare gene expression profiles between healthy and diseased gingival tissues on a cell type-specific level. GO enrichment analysis reveals potential activation of epithelial cells in PD tissues, as indicated by the enrichment of GO terms related to growth factor receptor binding and epidermal growth factor receptor binding (Fig. 5e). Conversely, B cells in PD tissues exhibit enrichment of GO terms associated with B cell activation, suggesting an active immune response (Fig. 5f). By sub-clustering B cells, we identify four B cell subtypes, including memory B, activated memory B, immature B and plasma, and it shows that antibody-produced plasma cell has highest proportion (Fig. 6a and b). Pseudotime analysis results is consistent with B cell differentiation process (Fig. 6c). These results suggest that a stable immune system response is already established in periodontitis tissue.

### Features of Shaoxia platform

Beside GUI feature, Shaoxia platform also has some other useful features. Firstly, Shaoxia enable interactive response to cell quality control, dimension reduction, cell clustering and cell type annotation. As is well known, these analysis steps involve numerous parameters that need to be set. For instance, varying the parameters for quality control can yield different results, and trying different cell markers is necessary for annotating cell types. Shaoxia can present the results of these steps immediately as users modify the parameters. This allows users to adjust the parameters of these steps until they achieve the best results. Secondly, to address “big data” problem of scRNA-seq data analysis, Shaoxia doesn’t save the intermediate files which generated during the data processing. In contrast, we design a special MySQL database for Shaoxia to save parameters of each analysis that set by user. This design will save a lot of hard disk space if users install Shaoxia and use it on their own computer systems. Thirdly, Shaoxia is a multi-user analysis platform, it allows multiple users to login and use it at the same time, thus it makes teamwork easier. Fourthly, Shaoxia can handle different type of input data, including FASTQ file generated by 10X Genomics, ZIP file of cellranger software results and TXT file of expression matrix, thus has a very high degree of flexibility. Last but not the least, Shaoxia has a task scheduling system at backend, it can schedule downstream analysis jobs on the HPC system or a single computer server. Therefore, Shaoxia can release the total power of modern compute systems to accelerate the analysis of scRNA-seq data.

Compared to previously published analysis platform, Shaoxia has several advantages. Firstly, it has rich downstream analyses. SC1 [25], ICARUS [26], and SingleCAnalyzer [27] are analytics platforms that have been published in recent years, all of them just provides enrichment analyses for scRNA-seq data. Therefore, unlike Shaoxia, they may not satisfy the needs of most of researchers. Secondly, Shaoxia supports multiple input data types, while SC1 [25] does not support FASTQ file and ICARUS [26] only allow FASTQ files as input. Thirdly, all three of them does not have an analysis framework, but a clear framework could help researchers get a better understanding of scRNA-seq data analysis procedure. Finally, Shaoxia boasts superior interaction design, particularly in upstream analysis and cell type annotation.

While Shaoxia is a powerful tool, it currently focuses on analyzing scRNA-seq data. To expand its capabilities, future versions will incorporate analysis pipelines for other single-cell sequencing methods like scATAC-seq, transforming Shaoxia into a comprehensive single-cell omics data analysis platform. Additionally, Shaoxia is a continuously evolving platform, and it can benefit from integration with other valuable software tools. Tools like GPTCelltype [28], SingleR [29], and scType [30] could provide helpful references for manual cell-type annotation. Integrating SCUBI [31], Palo [32], and findPC [33] could enhance cell clustering analysis, while Slingshot [34] and TSCAN [35] would offer more flexibility for pseudotime analysis. Besides, Shaoxia's response time may be affected by large datasets and network issues, leading to high latency. To address this, installing and using Shaoxia in a local network environment is recommended. By using Shaoxia, researchers can get rid of learning several programing languages (which may be very hard for some wet-lab researchers) or writing repetitive code and obtain rich analysis results from their data easily.

## Conclusion

scRNA-seq has revolutionized the field of genomics by allowing researchers to explore the heterogeneity of gene expression at the individual cell level. This powerful technology provides unprecedented insights into cellular diversity and function. However, the sheer volume and complexity of scRNA-seq data and tedious bioinformatic pipelines necessitate sophisticated and interactive analysis platforms to extract meaningful biological information. Toward this end, we introduce Shaoxia, empowering researchers with a comprehensive toolkit for extracting meaningful biological insights from single-cell transcriptomic data and automating the analysis pipeline, making it accessible to researchers across diverse biological disciplines. Shaoxia represents a good open-source analysis software for researchers who use scRNA-seq technology to interrogate the essential biological issues, and we believe that it can serve as a foundational analysis platform for scRNA-seq data.

## Availability and requirements

Project name: Shaoxia.

Project home page: https://github.com/WiedenWei/shaoxia.

Operating system(s): Linux (for server side), cross-platform (for user side).

Programming language: Python, R, Typescript, Shell.

Other requirements: Nginx, Slurm, Supervisor.

License: GNU General Public License version 3.

Any restrictions to use by non-academics: None.

### Supplementary Information

**Supplementary Material 1**.

**Supplementary Material 2**.

**Supplementary Material 3**.

## Data Availability

The scRNA-seq dataset of PBMCs is publicly available and can be downloaded using this link (http://s3-us-west-2.amazonaws.com/10x.files/samples/cell-exp/2.1.0/pbmc8k/pbmc8k_fastqs.tar). The scRNA-seq dataset of oral tissue can be downloaded via the following link: https://www.ncbi.nlm.nih.gov/geo/query/acc.cgi?acc=GSE164241.

## Abbreviations

scRNA-seq: Single-cell RNA sequencing
COVID-19: Corona virus disease 2019
scATAC-seq: Single-cell sequencing assay for transposase-accessible chromatin
HPC: High performance compute
PBMC: Peripheral blood mononuclear cell
DEGs: Differentially expressed genes
KEGG: Kyoto encyclopedia of genes and genomes
GO: Gene ontology
GSEA: Gene set enrichment analysis
GSVA: Gene set variation analysis
GUI: Graphical user interface
QC: Quality control
DC: Dendritic cell
NK: Natural killer cell
NKT: Natural killer T cell

## References

[1] Hwang B, Lee JH, Bang D (2018). Single-cell RNA sequencing technologies and bioinformatics pipelines. Exp Mol Med.

[2] Naulaerts S, Datsi A, Borras DM (2023). Multiomics and spatial mapping characterizes human CD8+ T cell states in cancer. Sci Transl Med..

[3] Zhang JY, Wang XM, Xing X (2020). Single-cell landscape of immunological responses in patients with COVID-19. Nat Immunol.

[4] Williams DW, Greenwell-Wild T, Brenchley L (2021). Human oral mucosa cell atlas reveals a stromal-neutrophil axis regulating tissue immunity. Cell..

[5] Zhou F, Wang R, Yuan P (2019). Reconstituting the transcriptome and DNA methylome landscapes of human implantation. Nature.

[6] Han X, Zhou Z, Fei L (2020). Construction of a human cell landscape at single-cell level. Nature.

[7] Kim N, Kim HK, Lee K (2020). Single-cell RNA sequencing demonstrates the molecular and cellular reprogramming of metastatic lung adenocarcinoma. Nat Commun.

[8] Anderson D, Skut P, Hughes AM (2020). The bone marrow microenvironment of pre-B acute lymphoblastic leukemia at single-cell resolution. Sci Rep.

[9] Chen Z, Zhou L, Liu L (2020). Single-cell RNA sequencing highlights the role of inflammatory cancer-associated fibroblasts in bladder urothelial carcinoma. Nat Commun.

[10] Baek S, Lee I (2020). Single-cell ATAC sequencing analysis: From data preprocessing to hypothesis generation. Comput Struct Biotechnol J.

[11] Satija R, Farrell JA, Gennert D (2015). Spatial reconstruction of single-cell gene expression data. Nat Biotechnol.

[12] Vento-Tormo R, Efremova M, Botting RA (2018). Single-cell reconstruction of the early maternal–fetal interface in humans. Nature.

[13] Aibar S, González-Blas CB, Moerman T (2017). SCENIC: single-cell regulatory network inference and clustering. Nat Methods.

[14] Clarke ZA, Andrews TS, Atif J (2021). Tutorial: guidelines for annotating single-cell transcriptomic maps using automated and manual methods. Nat Protoc.

[15] Hu C, Li T, Xu Y (2023). Cell Marker 2.0: an updated database of manually curated cell markers in human/mouse and web tools based on scRNA-seq data. Nucleic Acids Res..

[16] Danecek P, Bonfield J K, Liddle J, et al. Twelve years of SAMtools and BCFtools. Gigascience. 2021;10(2):giab008. 10.1093/gigascience/giab008 PMC7931819 33590861

[17] Bergen V, Lange M, Peidli S (2020). Generalizing RNA velocity to transient cell states through dynamical modeling. Nat Biotechnol.

[18] Puram SV, Tirosh I, Parikh AS (2017). Single-cell transcriptomic analysis of primary and metastatic tumor ecosystems in head and neck cancer. Cell.

[19] Gu Z (2022). Complex heatmap visualization iMeta.

[20] Wickham H (2011). ggplot2. Wiley interdisciplinary reviews: computational statistics.

[21] Wu T, Hu E, Xu S (2021). clusterProfiler 4.0: A universal enrichment tool for interpreting omics data. Innovation (Camb)..

[22] Hänzelmann S, Castelo R, Guinney J (2013). GSVA: gene set variation analysis for microarray and RNA-seq data. BMC Bioinformatics.

[23] Subramanian A, Tamayo P, Mootha VK (2005). Gene set enrichment analysis: a knowledge-based approach for interpreting genome-wide expression profiles. Proc Natl Acad Sci.

[24] Trapnell C, Cacchiarelli D, Grimsby J (2014). The dynamics and regulators of cell fate decisions are revealed by pseudotemporal ordering of single cells. Nat Biotechnol.

[25] Moussa M, Măndoiu II. SC1: a tool for interactive web-based single-cell RNA-Seq data analysis. J Comput Biol. 2021;28(8):820–41. 10.1089/cmb.2021.0051 34115950

[26] Jiang A, Lehnert K, You L, et al. ICARUS, an interactive web server for single cell RNA-seq analysis. Nucleic Acids Res. 2022;50(W1):W427–33. 10.1093/nar/gkac322 PMC9252722 35536286

[27] Prieto C, Barrios D, Villaverde A. SingleCAnalyzer: interactive analysis of single cell RNA-Seq data on the cloud. Front Bioinform. 2022;2:793309. 10.3389/fbinf.2022.793309 PMC9580930 36304292

[28] Hou W, Ji Z. Assessing GPT-4 for cell type annotation in single-cell RNA-seq analysis. Nat Methods. 2024. 10.1038/s41592-024-02235-4. 10.1038/s41592-024-02235-4 PMC11310073 38528186

[29] Aran D, Looney A P, Liu L, et al. Reference-based analysis of lung single-cell sequencing reveals a transitional profibrotic macrophage. Nat Immunol. 2019;20(2):163–72. 10.1038/s41590-018-0276-y PMC6340744 30643263

[30] Ianevski A, Giri A K, Aittokallio T. Fully-automated and ultra-fast cell-type identification using specific marker combinations from single-cell transcriptomic data. Nat Commun. 2022;13(1):1246. 10.1038/s41467-022-28803-w PMC8913782 35273156

[31] Hou W, Ji Z. Unbiased visualization of single-cell genomic data with scubi. Cell Reports Methods. 2022;2(1). 10.1016/j.crmeth.2021.100135 PMC8871596 35224531

[32] Hou W, Ji Z. Palo: spatially aware color palette optimization for single-cell and spatial data. Bioinformatics. 2022;38(14):3654–6. 10.1093/bioinformatics/btac368 PMC9272793 35642896

[33] Zhuang H, Wang H, Ji Z. findPC: An R package to automatically select the number of principal components in single-cell analysis. Bioinformatics. 2022;38(10):2949–51. 10.1093/bioinformatics/btac235 35561205

[34] Street K, Risso D, Fletcher RB, et al. Slingshot: cell lineage and pseudotime inference for single-cell transcriptomics. BMC Genom. 2018;19:1–16. 10.1186/s12864-018-4772-0 PMC6007078 29914354

[35] Ji Z, Ji H. TSCAN: Pseudo-time reconstruction and evaluation in single-cell RNA-seq analysis. Nucleic Acids Res. 2016;44(13):e117. 10.1093/nar/gkw430 PMC4994863 27179027

