# Supplementary figures and images for: Shaoxia: a web-based interactive analysis platform for single cell RNA sequencing data
Source: BMC Genomics. 2024 Apr 24;25:402. doi: 10.1186/s12864-024-10322-1 (PMC11040744; doi:10.1186/s12864-024-10322-1)

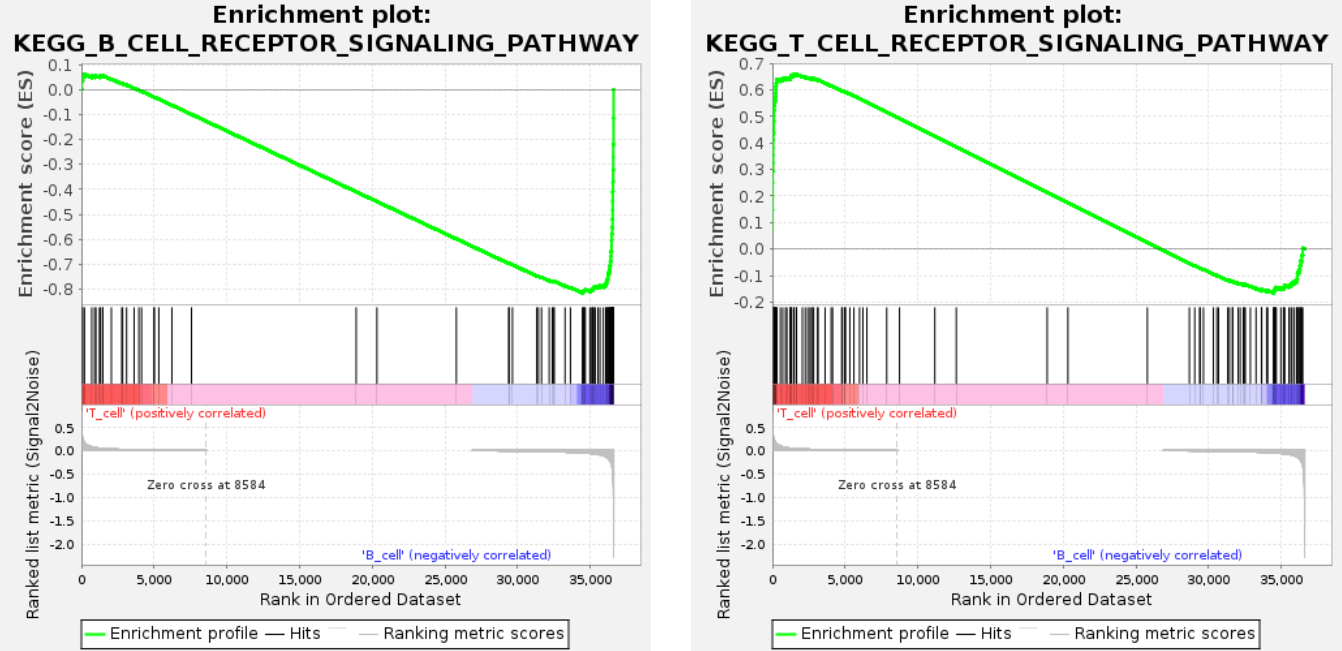


Figure S1. GSEA results of expression data of T cell and B cell.

Supplement: Supplementary file 1 — Supplementary Material 1. [file 12864_2024_10322_MOESM1_ESM.docx]

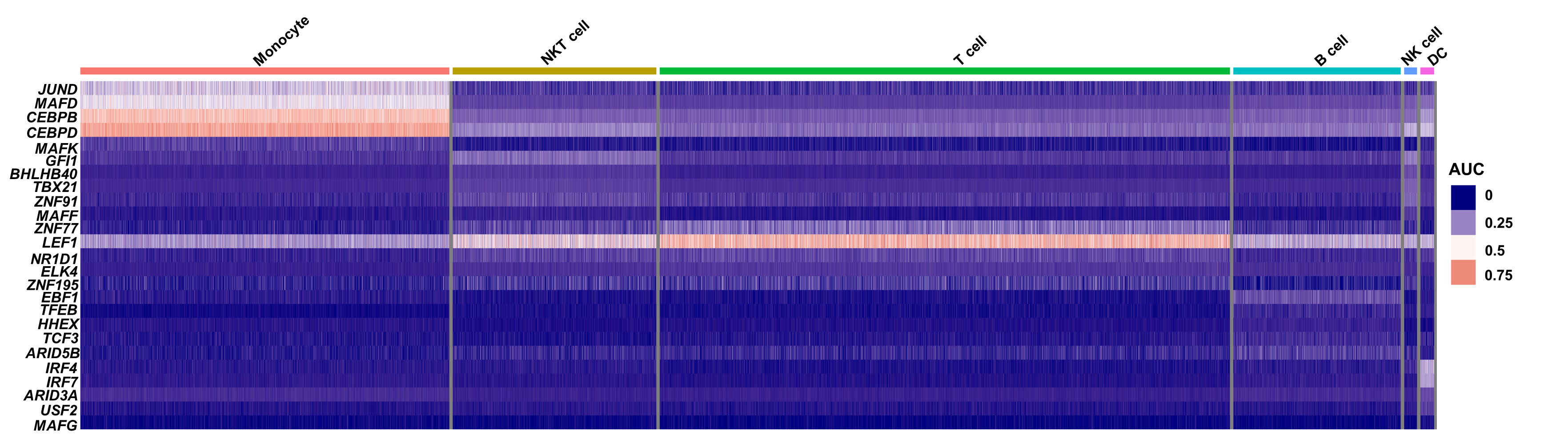
 Figure S3. Gene regulation analysis result of scRNA-seq data of PBMCs.

Supplement: Supplementary file 3 — Supplementary Material 3. [file 12864_2024_10322_MOESM3_ESM.docx]
