# Supplementary material for: Shaoxia: a web-based interactive analysis platform for single cell RNA sequencing data
Source: BMC Genomics. 2024 Apr 24;25:402. doi: 10.1186/s12864-024-10322-1 (PMC11040744; doi:10.1186/s12864-024-10322-1)
Supplement: Supplementary file 2 — Supplementary Material 2. [file 12864_2024_10322_MOESM2_ESM.docx]

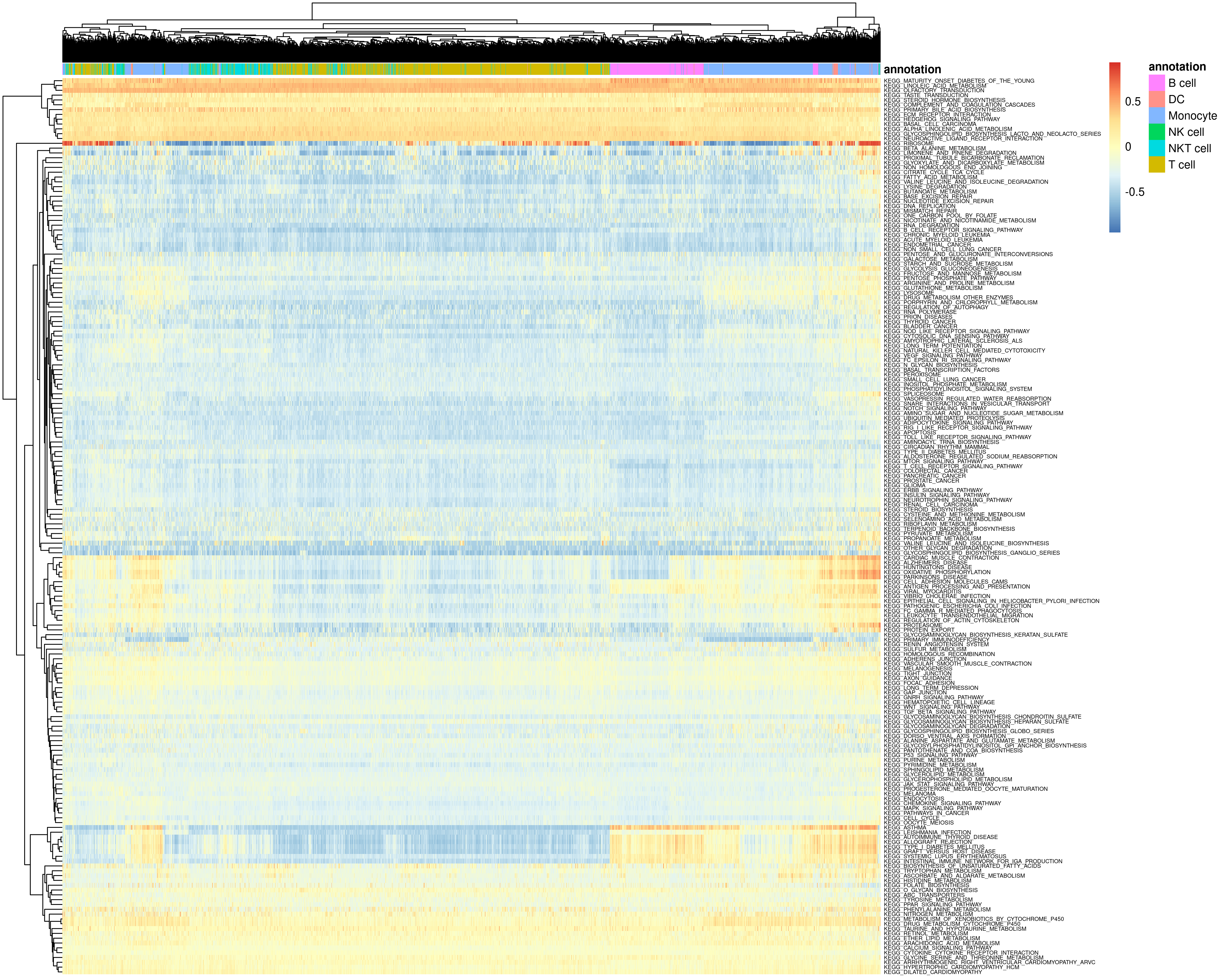
Figure S2. GSVA result of scRNA-seq data of PBMCs. **The red arrow-indicated pathways are the B cell and T cell receptor signaling pathways.**
